# Self-Perceived Employability of Workers With Disability: A Case Study in an Educational Farm

**DOI:** 10.3389/fpsyg.2022.871616

**Published:** 2022-06-13

**Authors:** Stefania Fantinelli, Teresa Di Fiore, Alessia Marzuoli, Teresa Galanti

**Affiliations:** ^1^Department of Humanities, University of Foggia, Foggia, Italy; ^2^Department of Psychological, Health and Territorial Sciences, G. d’Annunzio University of Chieti and Pescara, Chieti, Italy

**Keywords:** self-empowerment, employability, inclusion, disability, social purpose

## Abstract

**Background:**

The job placement of persons with disability is often threatened by prejudices and stereotypes; even when they are employed, they have less qualified and less paid jobs. The aim of this study was to investigate the self-perceived employability in a sample of workers with disability, hypothesizing to find a good level of self-determination and positive meaning of work.

**Materials and Methods:**

Ten semi-structured interviews have been conducted, applying a mix-method to the data analysis through qualitative interpretation and quantitative content analysis. Results confirmed what recent literature shows about the need for job inclusiveness for persons with disability and also underlined a profound sense of satisfaction related to the job, strong identity, and empowerment derived from the job involvement.

**Conclusion:**

Practical implications are related to the job design procedure inspired by diversity management, in order to pay attention to every single diversity and ensure equity and inclusion.

## Introduction

Work is considered one of the most important aspects in one’s life, in particular for the positive impact it has in terms of autonomy and independence. In fact, it is not just a mere practice that allows people to provide financial support, but an important educational activity that contributes to increase the level of social participation and the affirmation of a more emancipated self-image (Caldin and Scollo, 2018). However, this assumption is as true as not universally valid, especially if we think about people with disabilities (PWDs).

For this reason, this study aimed to underline the urgency of Disability Management inspired by the social purpose of equity and inclusion that is able to engage PWDs in working activities and promote their perception of employability.

The right to the employability of PWDs, to which it refers also Article 27 of the ONU Convention on the Rights of Person with Disabilities (2006), clashes with many difficulties and physical but mostly cultural obstacles. In Italy, despite the advantages and benefits of hiring workers with disabilities guaranteed by Law 68/99, employers still tend to consider disability a problem for productivity (Istituto Nazionale di Statistica [ISTAT], 2019). At European Union (EU) level, only 50.6% of persons with disabilities are employed, compared with 74.8% of persons without disabilities (Nancy, 2017). Moreover, the last country report of the Academic Network of European Disability experts reveals that women with disabilities, young disabled persons, and persons with high support needs are more likely to be discriminated against and excluded from the labor market (Grammenos, 2017).

Paradoxically, recent studies underlined the positive consequences for organizations that hire PWDs, in terms of productivity, loyalty, and lower turnover intentions. In particular, a study by Houtenville and Kalargyrou (2012) showed that organizations tend to recruit PWDs to increase retention and productivity; similarly, Hernandez and McDonald (2010) argued that employees with disabilities seem to be more loyal to their own organization than employees without disabilities. Likewise, a study by Romano (2003) on turnover intention in a call center revealed a lower turnover rate (approximately 8%) among PWDs than an average rate of 45%.

Moreover, job inclusion of PWDs seems to be a strategic decision also in terms of Employer Branding because it is able to improve the image of the company, as well as of the business. A positive work environment is often associated with a more fruitful commitment of workers (Friso and Scollo, 2018). Therefore, the need to support and encourage a social and cultural inclusion-oriented approach that is able to promote autonomy and participation of PWDs in the work world is evident.

However, progress toward comprehensive inclusive employment is hampered by numerous barriers. The first one is represented by the substantial differences in perceptions of the employers and employees regarding the positive benefits of employing PWDs. According to Ramachandra et al. (2017), some of the barriers faced by the employees with disabilities included lack of physical access to the worksite and within the worksite; communication and information barriers; and lack of training opportunities. A study by Saunders and Nedelec (2014) underlined that PWDs view employment as a means of expressing their worth in a society, which places barriers in their path both for education as well as for employment opportunities.

In the following paragraphs, the conceptual framework of this study will be presented in more detail.

### Disability Management

Since ancient times, the disability has been approached with a real negative attitude: according to Plutarco, children with impairments were abandoned under the Taigeto mountain; after 20 centuries, the Norimberga laws stated that people with physical or mental disability could be sterilized. It is only after World War II that PWDs are recognized as persons with rights, other than persons who need care (Soresi, 2016).

In the field of organizational research, disability has been studied from various perspectives. The first one is related to a medical model of disability that defines disabled people in terms of biological properties (Colella, 2001). According to this perspective, the main interest of researchers is to detect, avoid, and categorize disabilities, and to help and rehabilitate PWDs through medical or psychological treatments. The risk of this theoretical approach is to foster stigma and prejudices on disabilities in the mind of employers, especially regarding the minor productivity of PWDs.

In contrast, the second perspective is related to a social model, according to which disability is a social creation (Oliver, 1996) and it can be easily resolved by removing barriers in the social and material environments. Researchers that start from this point of view are interested in the discrimination and the physical and social structures that inhibit and exclude PWDs from organizational life (Abberley, 2002). The drift of the social model is the victimization of PWDs and the carelessness of the lived experiences.

A determinant turning point is the ONU Convention in (2006), it represents the first attempt to unify the idea of a person to the idea of a human being, and in the article 1 comma 2, there is an important definition: “persons with disabilities include those who have long-term physical, mental, intellectual or sensory impairments which in interaction with various barriers may hinder their full and effective participation in society on an equal basis with others.”

Later, even scientific literature defines disability as a construct derived from the social and physical contexts, social supports, or architectural barriers that contribute to determine the social condition of the person, who will be defined as limited or unable (Altman, 2014). Thus, the definition of disability is 2-fold as follows: there is a strictly personal-individual dimension and a social-contextual dimension; they are both relevant in a work setting.

However, this is the perspective of Diversity Management and, in particular, of Disability Management, a school of thought that aims to build a highly inclusive working environment, which is able to guarantee even workers with disabilities a significant space for personal growth, autonomy, and self-determination.

Several studies discussed the relationship between diversity and performance of an organization. At an individual level, some researchers explore the link between diversity management and individual outcomes such as commitment, absenteeism, satisfaction, and turnover (Tsui et al., 1992); instead, at an organizational level, researchers investigated outcomes such as performance, productivity, and firm competitiveness (Richard, 2000). Some researchers have found that diversity management positively influences organizational effectiveness, firm performance, and productivity (Watson et al., 1993; Richard et al., 2004). Similarly, Horwitz and Horwitz (2007) found that job-oriented diversity has a positive impact on team performance.

As Narayanan and Terris (2020) argued, individuals with disabilities are a vital part of any economy and an important source of talent, and they are too often ignored. Moreover, improving productivity can result in greater commercial viability for enterprises and, at the same time, can help their social mission by supporting individuals with disabilities (Neumann, 2019). According to Hoffman (2013), organizations should have the role of integrating individuals with disabilities into equal opportunity environments.

However, according to the ONU report about disability and development (United Nations [UN], 2018), there are more than 1 billion PWDs that are not in line with the sustainable development goals of the Agenda ONU 2030, and one of the main topics of these goals is the promotion of the empowerment.

### Psychological Empowerment

In the past three decades, positive psychology (Seligman and Csikszentmihalyi, 2014) focused attention on the construct of psychological empowerment (PE) (Zimmerman and Rappaport, 1988). It includes beliefs about one’s competence, efforts to exert control, and an understanding of the socio-political environment. The latter aspect also includes the ability to identify both individual resources and the factors that influence decision-making processes. According to Zimmerman (1995), there are three different dimensions of PE: intrapersonal, interactional, and behavioral components. The intrapersonal components include locus of control, self-efficacy, and motivational aspect of perceived control (Zimmerman and Rappaport, 1988); the interactional components refer to the ability to use problem-solving to influence the environment. Finally, the behavioral components refer to exert control in community organizations or activities. Applied to PWDs, empowerment can be seen as the possibility to have the same degree of control over one’s life and the conditions that affect it as is generally enjoyed by PWDs (Harp, 1994). However, the theory of empowerment has a limited impact on individuals with traditional disabilities because it promotes temporary interventions that are not able to produce durable effects.

Nevertheless, the stigma associated with disabilities is the main obstacle to the empowerment process (Dervishi, 2013); prejudice and discrimination can hinder the job placement of persons with disability (Soresi, 2016); in fact, there is the common idea that disability is incompatible with productivity. Opposite to this prejudice, there are data that highlight how the exclusion of PWDs from the workforce has costs for society, overall in terms of their productive potential (Calderón-Milán et al., 2020). Other studies confirmed that people with intellectual disability can be successfully involved in the process of start-up a company, with also benefits the social relations (Barba-Sánchez et al., 2021).

### Perceived Employability

The literature provides several definitions for employability, and as a theoretical frame for this study, we take into consideration the model elaborated by Fugate et al. (2004) because it embraces all the dimensions described by other authors and it represents a sort of reference point about this theme. Employability can be defined as a psycho-social multidimensional construct focused on the individual and on the personal proactivity; it is the outcome of three main factors as follows: career identity, personal adaptability, and social and human capital (Fugate et al., 2004). In the modern work context, workers can directly manage their own careers, being proactive and flexible, and they can improve their job positions in combination with the context demands (Fugate et al., 2004). In this term, employability represents a great coping resource, also in situations of job search even after dismissal.

The focus on personal resources particularly implied in a job search process contributes to the definition of the perceived employability as follows: it is related to the individual beliefs regarding the opportunities to find a new job (Berntson, 2008).

Another relevant job dimension in terms of identity, wellbeing, and values is the meaning of work, defined as the balance between the personal characteristics of workers and their expectations. Meaning of work has also been related to perceived empowerment (Spreitzer, 1995).

Four main sources of meaning have been identified in literature as follows: the self, the other persons, the work context, the national culture, and the spiritual life (Rosso et al., 2010). Values and meanings associated with work are personal resources useful to guide choices and to better understand if a job experience is in line with one’s own beliefs and expectations (Berings et al., 2004). Several authors investigated the impact of the meaning of work on different organizational variables, and it has been confirmed that the meaningfulness of work positively affects job performance; furthermore, the perceived organizational support increases employees’ meaning of work (Akgunduz et al., 2018).

In the actual work context, persons with disability have often less qualified and less paid tasks (Schur and Kruse, 2002; Meager and Higgins, 2011) due to their levels of education and ability (Greve, 2009; Mitra and Sambamoorthi, 2014); moreover, employers doubt about competencies of workers with disabilities, and they expect more deviant behaviors and safety rule refusal (Ju et al., 2013). Other scholars investigated the employability attitudes of graduate persons with disability; in particular, they focused on expectations, perceived employability, resilience, and self-empowerment (Magrin et al., 2019). Notably, 60% of persons with disability defined work as an important opportunity for personal growth, compared with 40% of people without disability. Furthermore, graduate people showed a higher level of resilience and self-empowerment that indirectly can promote the perceived employability.

A significant number of studies have been conducted over the past three decades, highlighting the capacity and capability of disabled people to perform complex work tasks and including those with extensive disability (Agran et al., 2014). In contrast, to date, almost all disability-specific workplace literature has focused on barriers to employment, with less focus on career success (Baldridge and Kulkarni, 2017).

Moreover, even if some research shows that having a disability is a barrier to securing and maintaining employment (Lindsay et al., 2015), less is known about specific factors that contribute to these poor outcomes, and even less is known about factors and processes that might improve them. Finally, there is scarce literature that considers the direct point of view of workers with disability (Strindlund et al., 2018).

### Aim of This Study

Starting from these assumptions, the aim of this case study was to investigate the perceived employability and the meaning of work in a sample of persons with disability, to better underline (1) the characteristics of disabled workers in terms of expectations and attitudes toward work, perceived employability, and perceived impact of disability on their career, as well as in terms of levels of resilience resources and employability resources and (2) the potential resources for preventing perceived negative impact of disability on employment opportunities and promoting the perception of employability.

## Materials and Methods

To provide the research with a thorough structure and quality, the Standard for Reporting Qualitative Research (SRQR–O’Brien et al., 2014) was followed.

### Setting and Sample

The educational farm represents a form of inclusiveness where PWDs can have an active role in the promotion of their wellbeing, rather than just being passive receivers. The participants were recruited, thanks to the collaboration within an educational farm that currently employs around 30 persons with disability. We defined a few inclusion criteria as follows: persons with a form of intellectual disability, who was employed at the time of the data collection, and who manifested interest in participating; the data were collected between September and October 2019. The sample (Table 1) is quite small, and there are 10 participants, five women and five men aged between 23 and 43 years, and the mean age is 32.6 years (SD: 5.89); age is not available for one participant. A total of 66.66% of samples have high school graduation, and 33.33% of samples have middle school graduation (educational qualification is not available for one participant). The majority of them (8) have Down syndrome, and some have other syndromes with mild or medium mental disability. All the participants live with their family and nobody is married.

**TABLE 1:** Description of the sample.

Participant	Sex	Age	Type of disability	Syndrome	Educational qualification	Marital status
1	M	33	Congenital	West syndrome	High school graduation	Unmarried
2	M	32	Congenital	Down syndrome	High school graduation	Unmarried
3	F	36	Congenital	Cognitive delay	High school graduation	Unmarried
4	M	29	Congenital	Down syndrome	–	Unmarried
5	F	27	Congenital	Down syndrome	High school graduation	Unmarried
6	F	34	Congenital	Down syndrome	Middle school graduation	Unmarried
7	M	43	Congenital	Down syndrome	Middle school graduation	Unmarried
8	F	–	Congenital	Down syndrome	High school graduation	Unmarried
9	M	37	Congenital	Down syndrome	High school graduation	Unmarried
10	F	23	Congenital	Down syndrome	Middle school graduation	Unmarried

The researcher who interviewed the participants had a 10-year previous experience in the field of disability, which helped and stimulated the interviewees, pushing them to be reflective on the topics covered, addressing the topics of the interview with the right degree of depth, and connecting the several ideas emerged from the real experiences of the interviewees.

The interviews lasted approximately 50 min each, and informed consent has been signed by the parents or carers; all the interviews were recorded and transcribed verbatim in order to facilitate the interpretation analysis, which has been carried out by two independent researchers in order to preserve the reliability of the interviews. The research conforms to the provisions of the Declaration of Helsinki in 1995 (as revised in Edinburgh 2000), and all ethical guidelines were followed as required for conducting human research, including adherence to the legal requirements of the study countries. According to the Italian Association of Psychology (AIP), at the time of this study, a very general document was available, whose guidelines we followed.

### Materials

The interview (Appendix A) was semi-structured and in line with the theoretical principles of the narrative interview in order to offer the researcher direct access to the cognitive world of participants and an understanding of their reality representations (Atkinson, 2002). According to Bruner (2002), stories represent our way of organizing, interpreting, and giving meaning to the experiences we live; every story provides the researcher with enough elements to know the person’s values and catch his or her Unicity (Atkinson, 2002).

### Methods

Our research design is inspired by the paradigm of *methodological appropriateness* (Patton, 1990), according to which a researcher should choose a collecting and analyzing data method consisting of the research object rather than with competencies possessed by the researcher. Thus, being the individual self-perceptions of workers with disability still partly unexplored, we adopted an intensive and explorative method in order to provide the research field with a new perspective and a new angle of observation.

It is through the language that participants can convey different meanings, for example, the use of metaphors or analogies can be a source of analytic strategy (Atkinson, 2002). The *grounded theory* (Glaser and Strauss, 1967) represents the theoretical framework of the method; according to Glaser and Strauss, the bottom-up analysis implies that row data suggest theories and hypotheses to researchers, making possible the creation of frequent clusters or categories relevant for the specific theme of the research.

Data have been analyzed both in qualitative terms (metaphors analysis and agentivity) and in quantitative terms (automatic content analysis supported by T-Lab software). Thus, the research aims to use a triangulation of methods, in order to guarantee scientific and methodological rigor (Denzin, 2012; Cortini, 2014). Triangulation, understood in this context as a crossing of distinct data analysis techniques, in fact, has the ability to preserve the heuristic power of the data collected at a qualitative level and the rigor of quantitative methods in order to protect their external validity. The choice of using triangulation for data analysis allows us to overcome the limits that have always characterized the two approaches. On the one hand, the quantitative method carries with it the risk of reducing the analysis of data to the pure processing of the same through the use of statistical software; on the other hand, qualitative methods do not have the ability to ensure sufficient validity that deviates significantly from here and now (Cortini, 2014; Cortini and Tria, 2014). For the qualitative part, a “classical” analysis of the discourse has been performed, consisting mainly of the analysis of metaphors, which is an indirect instrument useful to identify relevant elements that may influence the context. Metaphors can put a light on the unsaid, making it possible to infer thoughts or beliefs not directly expressed (Ervas et al., 2017).

The Metaphor Identification Procedure (Pitcher, 2013) allows a systematic approach to the identification and interpretation of metaphors, meant as personal illustrations of the reality and concrete images of the concept expressed (Pitcher, 2013). This explorative and bottom-up analysis implies a sort of complete openness to data, and the identification and categorization of metaphors were performed independently by two separate investigators, with subsequent accordance calculated, according to the Cohen kappa (0.87).

With regard to the quantitative analysis, we did a careful analysis of the content supported using the *T-Lab* software (analysis of occurrences and co-occurrences of words and Markovian sequences).

We have chosen to use the T-lab software to better adhere to the paradigm that inspired us while guaranteeing a qualitative analysis, relating to the analysis of the speech, and a quantitative analysis, relating to the analysis of the content (Cortini and Tria, 2014). Despite the typically qualitative nature of our data, which concerns transcripts of semi-structured interviews and therefore textual material, the T-lab software allowed us to carry out both types of analyses, namely, qualitative and quantitative, by triangulating the methods of analysis. According to SRQR (O’Brien et al., 2014), triangulation can enhance the trustworthiness and credibility of data analysis. The quantitative part, specifically, allowed us to identify the repetitions of words and the most frequent associations within the text.

## Results

### Metaphor Analysis

Two separate investigators performed the identification and categorization of metaphors independently, with subsequent accordance calculated, according to the Cohen kappa (0.87), and two relevant main clusters of metaphors were identified as follows: the first is related to self-empowerment and the second concerns the organizational climate. Table 2 shows the most relevant clusters of metaphors analyzed.

**TABLE 2:** Clusters of metaphors.

Cluster of metaphors	Example	Themes
Self empowerment	Ex 1. *They call me hurricane, where I go, the grass won’t grow*	Power, strength, determination
	Ex 2. *We have an extra gear*	
	Ex. 3. *I am a great teacher for the children*	Guide
Organizational climate	Ex. 4. *It is a family. I feel at home*	Satisfaction, gratitude

With regard to the first cluster, there are several examples that express the perceived self-empowerment:

Ex. 1: “They call me hurricane, where I go, the grass won’t grow.”
Ex. 2: “We have an extra gear.”
Ex. 3: “I am a great teacher for the children.”

These statements convey a strong self-efficacy and self-empowerment, the majority of participants are aware of their capabilities and skills, and this will in turn positively affect their perceived employability. In particular, the hurricane metaphor conveys a sense of power, strength, determination, and all qualities fostered by the supportive job environment. The identification with a teacher, in the third example, indicates that the person is aware to be a sort of guide for others.

The second cluster is the organizational climate; several participants declare that they would not look for another job because they are very satisfied with their current position.

Ex. 4: “It is a family, I feel at home.”

This statement has been enriched by another concept: participants are grateful to the organization because they have the possibility to improve themselves, thanks to job involvement. It is worthy to note this feeling of gratitude because, according to literature, gratitude in the workplace can foster both job performance and job satisfaction (Cortini et al., 2019) other than general wellbeing (Wood et al., 2010).

Except for the metaphors, there is another relevant theme frequently cited, and it is the self-determination attitude.

Ex. 5: “I feel fulfilled because I have a wage, I feel good about myself.”
Ex. 6: “I like the job, because I want to be more independent.”

With these declarations, it is possible to deduce that participants have elaborated a positive meaning of work that is relevant also in terms of identity since a lack of meaning can cause psychological deprivation and even depression (Steger et al., 2006; Tanno and Sakata, 2007).

In addition to the metaphor analysis, the agentivity has been analyzed; in particular, we observed the answers to the question “how did you choose your work.” The majority of participants used a passive form: “they call me,” and two persons talked about their active role in the choice, thanks to the help of the family. So it is possible to hypothesize that, according to our sample, persons with disability are quite often guided by other socially relevant persons, and the family seems to be the first place of influence.

### Content Analysis

To complete the analysis of the textual material collected through the interviews, we decided to run an automatic analysis of the content with the help of T-Lab software (Lancia, 2012; Cortini and Tria, 2014).

The preliminary step consisted of the combination of the transcripts of all interviews into one single “txt” format file, one after the other one. To perform the analysis with T-Lab, the text has been prepared with a disambiguation work, where homophonic words are distinct, and a lemmatization work, where different words are made to go back to a single lemmatic root, thus allowing an analysis of the conceptual content (Cortini and Tria, 2014). An example of disambiguation work is given by the *state* term, which can mean both “nation” and the past participle of the verb *to be* in Italian; disambiguating means to retrace in the text all the possible homophonic words and retag them.

With regard to the lemmatization process, which brings different linguistic forms to a common root, we take, as an example, the combination of the lemmas worker and working under the single lemma work; this operation is necessary to compute the concept of work in any of the linguistic forms with which it is expressed, and therefore, to combine all the ways, the times, and the people of the verb to work (a preliminary operation that the software performs automatically before starting the analyses), as well as all the other words used to evoke the concept of work. This analysis technique is defined as content analysis, and the intent is to analyze the conceptual content that is concealed behind apparently distinct linguistic forms.

### Association Analysis

After text preparation, the first automatic analysis of occurrences and co-occurrences was performed, and in technical jargon, it is defined as Association Analysis, and it was realized only on words with a frequency threshold of four times. We have decided to adopt the score of four as the cutoff of word occurrences (this means that we have run our analysis using only words that occur at least four times in the dataset), following the guidelines of the most recent papers related to automatic content analysis (Verrocchio et al., 2012; Cortini and Tria, 2014; Benevene et al., 2017).

**FIGURE 1:**
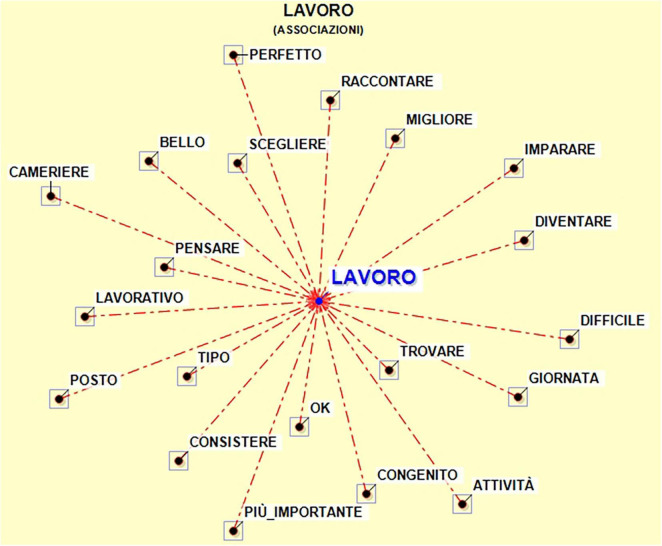
Associations with lemma “Lavoro” (work).

For what concerns the associations with the lemma work, it is possible to observe a graphical representation (Figure 1), where the more two words co-occur, the more they are close in dimensional space.

The co-occurrences are explained by the association index and the cosine coefficient, as can be seen in Table 3.

**TABLE 3:** Association index.

Lemma	Coeff.	C.E.(A)	C.E.(AB)	CHI^2^
To find	0.431	53	32	101,607
To choose	0.255	25	13	30,908
Better	0.225	25	13	30,908
To become	0.212	36	13	15,284
Consistency	0.198	12	7	19,839
Beautiful	0.196	25	10	14,457
Clever	0.151	42	10	3,526

*C.E.(A), occurrences; C.E.(AB), co-occurrences.*

According to our participants, the work is characterized by two big dimensions: the “consistency” (association index, Cosine 0.19) and the “beautiful” (association index, Cosine 0.19), confirming what is already noticed by the metaphor analysis. Moreover, it is interesting to note the associations with “become” (association index, Cosine 0.21) and “clever” (association index, Cosine 0.15), and they suggest that the work is requiring change and adaptation, said in different words, and flexibility. It seems that the reference to “clever” can be interpreted as the possibility to find themselves good at doing something, a clear indicator of self-efficacy and, in turn, a key factor of empowerment and employability.

## Discussion

There are several research studies, which seek to explore the employers’ point of view about disability, but very few studies involve persons with disability. A recent example is given by the qualitative research of Carmichael and Clarke (2020) as follows: they involved persons with disability with their caregivers and they deepened the meaning and the importance of work for participants. Our study seems to have reached similar conclusions as follows: work can be a source of independence and social support (Carmichael and Clarke, 2020), and according to our participants, work experience is synonymous with satisfaction and identity. All participants highlight the affective dimension of work, and the job produces joy, security, and belongings. Moreover, satisfaction is strictly related to motivation, and it can affect job performance. With regard to this latter dimension, it is also relevant to note that participants declare to have learned new skills and competencies during their job experience; in fact, a good learning climate can predict job performance (D’Alterio et al., 2019).

According to our results, the perceived employability received scarce attention from participants, and they stated that they would not look for another job and that they are satisfied with their current position. This statement can have a 2-fold interpretation as follows: it is an indicator of a strong organizational identity, and it can be a symptom of low perceived employability, as they do not hypothesize to look for a different job. Another explorative research investigated the relevant themes related to jobs for workers with disability (Akkerman et al., 2014); some themes that have been described are the participants’ perceived autonomy, opportunity for growth, and social connectedness. In line with these results, our participants stated that relational competencies are also crucial for wellbeing.

The practical implications of our research study appear to be different. First, supporting PWDs at work and promoting their inclusion is a way to enable them to contribute to society. Second, human resource practitioners could receive training inspired by the principles of disability management, meant as a proactive strategy oriented to remove those elements that make the job involvement for persons with disability hard (Geisen and Harder, 2011). However, literature highlighted some emerging trends that could hinder the employment of persons with intellectual disability as follows (Moore et al., 2017): the online recruitment process does not allow face-to-face interaction, where the recruiter could better investigate the individuals’ abilities. Furthermore, automation is often replacing roles suitable for persons with intellectual disability, such as routine tasks, thus limiting the opportunity for future employment (Moore et al., 2017).

Moreover, it can be confirmed that empowerment and self-determination should be the focus of every intervention directed to persons with disability, and there should be always an active involvement. A good job design can ensure the job placement of persons with disability, and diversity management is the practical application of this principle. So, specific attention is directed to individual differences in order to plan a widespread cultural change and tools or practices designed on single diversity.

The study has limitations. First, the convenience sample was taken from a single educational farm and it lacks significant external validity. Second, it could be found in the methodology; in fact, this research adopted a mix-method analysis on qualitative data collected on a small sample size, so that generalization of results is not applicable. However, this limitation must be underlined that it is quite difficult to reach persons with disability for scientific research. It is, in fact, a case study whose preliminary results, even if not representative, are definitely indicative of the urgency of future research.

Third, it could be found in the fact that this study considers only subjects with a single type of disability, moreover congenital. It would be interesting to also analyze acquired disabilities and physical disabilities, where the perception of self-efficacy could be very different.

Finally, this study considers only the point of view of the worker with a disability. If we look at disability as a multidimensional construct, which involves both personal and contextual factors, future research could investigate the social dimension of disability, intercepting first all families, employers, colleagues without disabilities, and clients.

## Conclusion

The very innovative point of our research is the direct involvement of workers with disability and their self-perception in terms of employability and meaning of work.

It seems that an appropriate conclusion should consider a 3-fold point of view as follows: psychological, social, and pedagogical dimensions. The psychological dimension is relevant because job employment can provide a positive and autonomous self-perception, other than a strong social identity for persons with disability. In fact, one of the main functions of work is to contribute to the definition of personal and social identity as well.

From the social point of view, disability should not be considered an individual attribute but a consequence of interactions among health conditions and personal and contextual factors, such as stereotypes and social barriers. In this perspective, the importance of a social purpose seems to be evident to promote the principles of equity and inclusion at all levels of society.

With regard to the pedagogical dimension, there are several levels: according to literature, persons with a disability could receive precise training on their occupational skills and employment awareness (Hitchings et al., 2001). It seems central to pay attention also to the family context, in particular, as far as we are concerned, there are very few research studies regarding the importance of necessary social support in order to increase the quality of life (both private and professional) of persons with disability. Finally, always from a pedagogical point of view, greater emphasis should be placed, in ministerial school curricula, on diversity education and inclusiveness, in order to nip in the bud prejudice and discrimination.

## Data Availability Statement

The raw data supporting the conclusions of this article will be made available by the authors, without undue reservation.

## Ethics Statement

Ethical review and approval was not required for the study on human participants in accordance with the local legislation and institutional requirements. The patients/participants provided their written informed consent to participate in this study.

## Author Contributions

SF, TD, and TG contributed to the conception and design of the study. AM organized the database. TD and SF performed the statistical analysis and wrote the first draft of the manuscript. TG wrote sections of the manuscript. All authors contributed to manuscript revision, read, and approved the submitted version.

## Conflict of Interest

The authors declare that the research was conducted in the absence of any commercial or financial relationships that could be construed as a potential conflict of interest.

## Publisher’s Note

All claims expressed in this article are solely those of the authors and do not necessarily represent those of their affiliated organizations, or those of the publisher, the editors and the reviewers. Any product that may be evaluated in this article, or claim that may be made by its manufacturer, is not guaranteed or endorsed by the publisher.

## Appendix A

### Interview Structure

1. Please describe your job; What are the tasks or the main activities?
2. Can you describe a typical day at work?
3. How did you choose your job?
4. What are the most difficult things that you have to cope with during the working day?
5. Can you remember some difficulties when you begin to work?
6. What kind of benefits do you experience during the work activities?
7. Does your organization promote workers’ autonomy and independence?
8. What is the most important thing that you have done to increase your professionality?
9. Considering your work experience, do you think you could find a different job in another organization?
10. If you would look for another job, do you think you will find a better job than now?
